# Temperature-dependent Raman spectroscopy and sensor applications of PtSe_2_ nanosheets synthesized by wet chemistry

**DOI:** 10.3762/bjnano.10.46

**Published:** 2019-02-13

**Authors:** Mahendra S Pawar, Dattatray J Late

**Affiliations:** 1 Physical and Materials Chemistry Division, CSIR-National Chemical Laboratory, Dr. Homi Bhabha Road, Pune 411008, India; 2 Academy of Scientific and Innovative Research (AcSIR), Ghaziabad 201002, India

**Keywords:** nanosheets, PtSe_2_, Raman spectroscopy, sensor, thermal effect

## Abstract

We report on a wet chemistry method used to grow PtSe_2_ nanosheets followed by thermal annealing. The SEM and TEM analysis confirms the formation of PtSe_2_ nanosheets. Furthermore, XRD, Raman, XPS and SAED patterns were used to analyze the crystal structure and to confirm the formation of the PtSe_2_ phase. The temperature-dependent Raman spectroscopy investigations were carried out on PtSe_2_ nanosheets deposited on Si substrates in the temperature range 100–506 K. The shifts in Raman active E_g_ and A_1g_ modes as a function of temperature were monitored. The temperature coefficient for both modes was calculated and was found to match well with the reported 2D transition metal dichalcogenides. A PtSe_2_ nanosheet-based sensor device was tested for its applicability as a humidity sensor and photodetector. The humidity sensor based on PtSe_2_ nanosheets showed an excellent recovery time of ≈5 s, indicating the great potential of PtSe_2_ for future sensor devices.

## Introduction

Graphene, the most well-studied example of the two-dimensional (2D) aromatic compounds, is the building block of all forms of carbon allotropes [1]. In recent years, it has been widely studied due to its extraordinary optical, electrical, mechanical, magnetic and chemical properties [2–5]. Like graphene and its organic analogues [6], inorganic 2D metal dichalcogenides also exhibit outstanding performance in many applications including transistors, sensors, photodetectors, solar cells, field emitters, battery materials, light harvesting and energy storage devices, catalyst for H_2_ generation, and drug delivery applications [7–12]. Most of the transition metal dichalcogenides (TMDCs) are semiconducting in nature with MX_2_ type – where M is a metal, M = W, Mo, Sn, Nb, V, etc. from group IV–V and X represents the chalcogenides family, X = S, Se, Te, etc. The metal atom M is sandwiched between layers of chalcogenide (X) atoms in the structure X–M–X. The TMDCs show diverse functional properties at the monolayer level in contrast to bulk due to the quantum confinement effect. Apart from this, these TMDCs, for example MoS_2_ and MoSe_2_, show an indirect to direct band gap transition [13–17].

A 2D platinum diselenide (PtSe_2_) material has recently joined the growing class of stable TMDCs due its promising applications. The 2D PtSe_2_ has not been explored much to date due to difficulties in synthesis. It is well known that bulk PtSe_2_ is a semimetal in nature with a nearly zero band gap [18–19]. With the help of theoretical calculations such as density functional theory (DFT) and local-density approximations (LDAs), it has been observed that bulk PtSe_2_ shows a semimetallic nature and single-layer PtSe_2_ has a semiconducting nature with a bandgap of 1.2 eV. Bilayer PtSe_2_ is also a semiconducting material but with a slightly smaller band gap than the monolayer material [19]. This layer-dependent conversion of semimetal-to-semiconductor transition has potential for electronic device applications [20–22]. Bulk PtSe_2_ was first prepared in 1909 by Minozzi from elements [23]. PtSe_2_ nanosheets have been recently prepared by heating thin foils of platinum in selenium vapors at 400 °C [19,24]. In this paper we have synthesized few-layer-thick PtSe_2_ nanosheets by a wet chemical method [25] at 90 °C using chloroplatinic acid (H_2_PtCl_6_) and Se powder as precursors followed by thermal annealing at 500 °C. Temperature-dependent Raman spectroscopic characterization was carried out on the materials.

## Materials and Methods

All the chemicals such as chloroplatinic acid, Se powder, hexamethylenetetramine, and NaBH_4_ were purchased from Sigma-Aldrich for the synthesis of PtSe_2_ nanosheets.

### Synthesis method

The PtSe_2_ nanosheets were synthesized using a solvothermal method followed by annealing at 500 °C using a previously described method for PtSe_2_ synthesis [25]. The PtSe_2_ material was prepared in two steps. The first step is the formation of the PtSe complex on the wall of a container by a wet chemical method; the second step is the phase transformation of PtSe_2_ by thermal annealing. 0.5 mL of a 0.015 M solution of H_2_PtCl_6_ was mixed with 0.5 mL of 0.5 M hexamethylenetetramine. In order to get a homogeneous solution, the mixture was carefully stirred for 15–20 s until the colour of the solution became slightly yellow; this is referred to as the Pt precursor. In another beaker 0.8 mg of Se powder was added into a 10 mL ice-cold solution of 0.1 M NaBH_4_ which acts as a strong reducing agent for the reduction of Se powder. The solution of Se was then heated in an oil bath at 90 °C for ≈20 min in order to completely reduce the Se. After complete reduction, the colour of the solution became dark brown and is referred to as the Se precursor. The Pt precursor was then slowly added into the Se precursor. The colour of the solution was found to suddenly change to greenish brown. The mixture was then kept undisturbed for ≈20 min. After 20 min the complex of Pt and Se was formed on the wall of the beaker. The complex was then washed several times using deionized water. First complex was transferred onto a Si substrate and heated at 100 °C on a hot plate. After complete evaporation, the substrate was annealed in a chemical vapour deposition system at 500 °C in argon gas atmosphere for 5 h. Supporting Information File 1, Figure S1 shows the schematic of the PtSe_2_ nanosheet synthesis steps.

### Sensor device fabrication and testing

Sensor devices were fabricated on a tin-doped indium oxide (ITO) substrate with a channel length of ≈300 µm and width ≈5 mm. The PtSe_2_ nanosheet powder was dispersed in *N*-methyl-2-pyrrolidone (NMP) solvent and then drop casted between the channels. The devices were further annealed in a vacuum furnace at 170 °C to improve the contact resistance and adhesion of the nanosheets with the substrate. The humidity sensing performance was investigated by exposing the sensor device to various relative humidity (RH) levels ranging from 11.3–97.3% as described in detail previously [26]. All of the electrical tests such as current–voltage (*I*–*V*) and current–time (*I*–*t*) measurements were carried out using a Keithley 2612A system source meter which was attached to a computer through a GPIB 488A interface. For the photodetection study, a green LED was used. All sensor experiments were carried out at ambient pressure and room temperature.

## Results and Discussion

### Structural characterization

**Figure 1:**
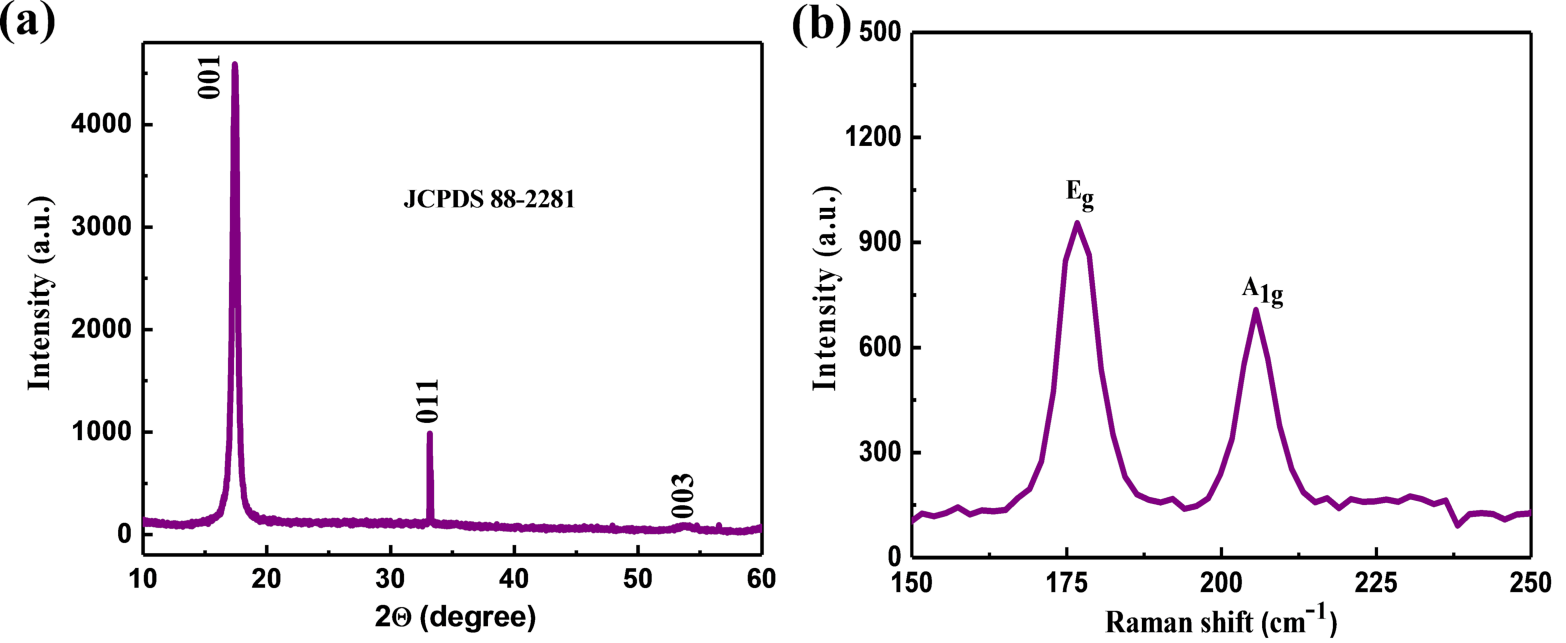
PtSe_2_ nanosheets. (a) Typical XRD pattern and (b) Raman spectra recorded at room temperature.

The structural characterization was carried out using X-ray diffraction (XRD) and Raman spectroscopy. Figure 1a shows the typical XRD pattern of the as-prepared sample deposited on a Si substrate. XRD was performed on a PANalytical X’pert pro dual goniometer diffractometer using Cu Kα radiation. The samples were mounted flat and scanned between 10 to 60°. The XRD pattern of the as-prepared sample shows the strong characteristic peaks around 2θ = 17.41° and 33.17° belonging to the (001) and (011) planes of PtSe_2_. These values match well with the JCPDS data card number (88-2281) and as observed in a previous report [27]. Figure 1b shows the Raman spectra of the as-prepared few-layer PtSe_2_ nanosheets. The Raman spectra were recorded using a Renishaw microscope at a wavelength of 532 nm with laser power ≈25 mW and laser spot diameter ≈1 µm. The typical Raman spectra recorded at room temperature consist of two distinct peaks, one at ≈176 cm^−1^ corresponding to the E_g_ mode and another slightly less intense peak at ≈205 cm^−1^ corresponding to the A_1g_ mode. The E_g_ mode in the Raman spectra corresponds to in-plane vibration due to the opposite motion of the upper and lower Se atoms. The A_1g_ mode in the Raman spectra corresponds to the out-of-plane vibration of Se atoms [22,28].

**Figure 2:**
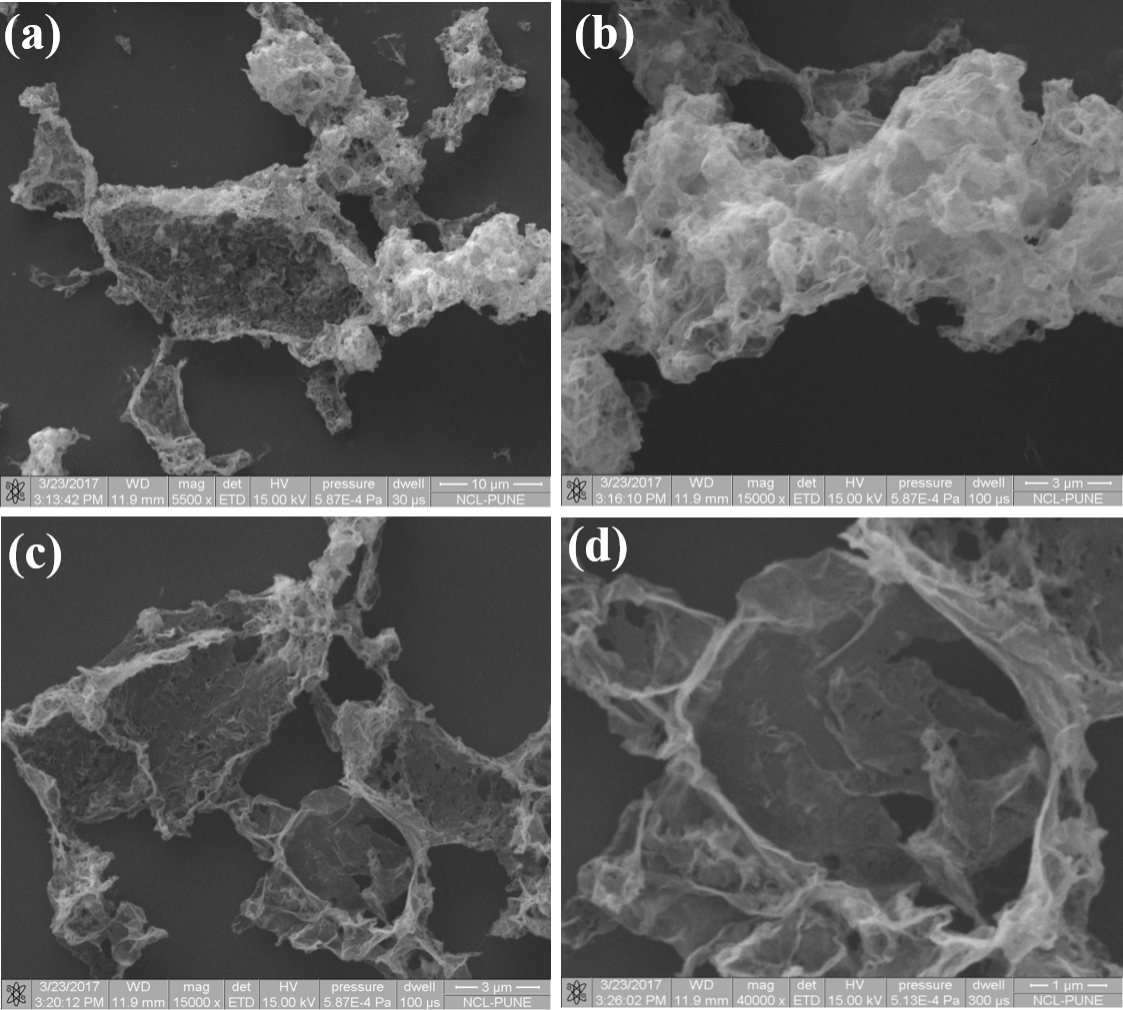
(a–d) Typical SEM images for PtSe_2_ nanosheets synthesized using the wet chemistry method.

**Figure 3:**
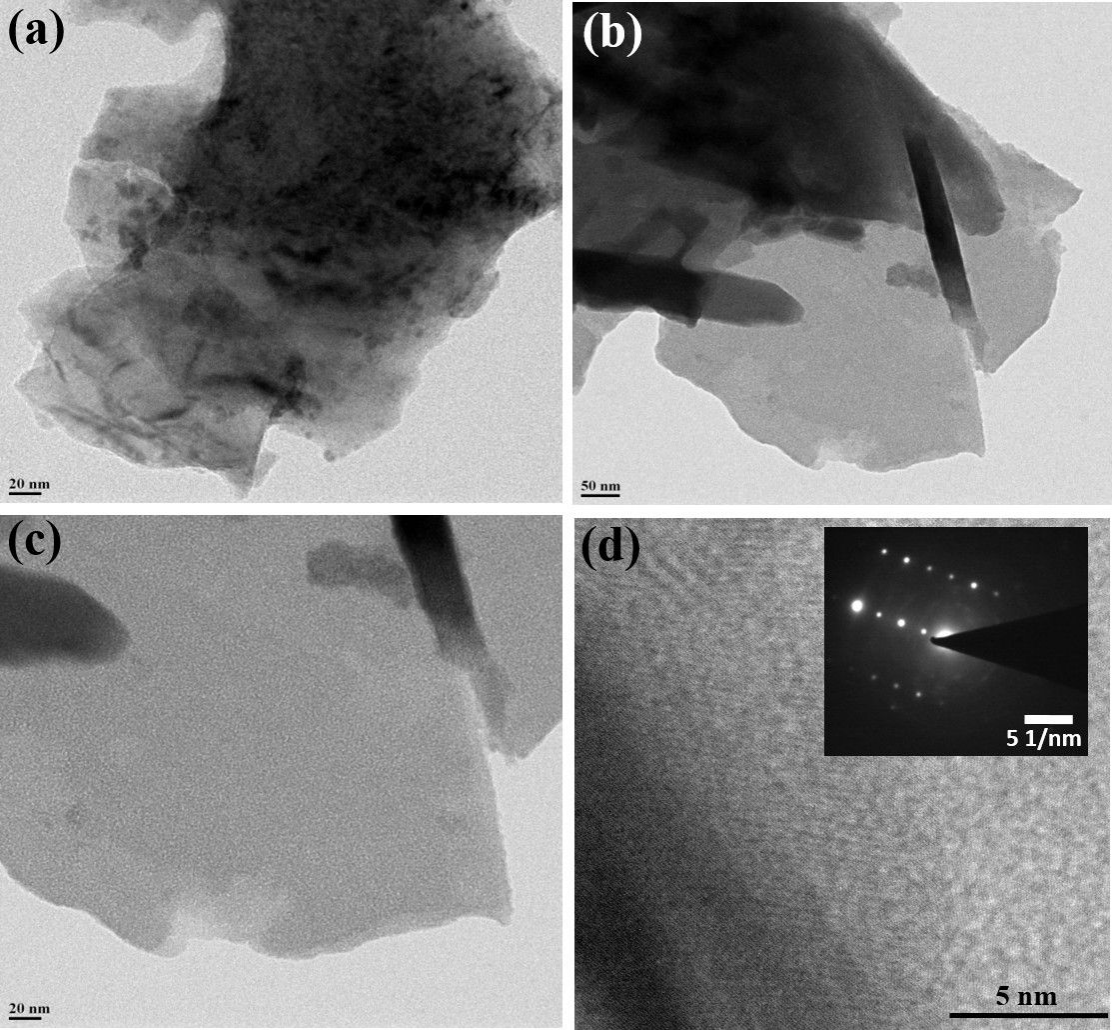
(a–c) Low-magnification TEM images and (d) a high-magnification TEM image, where the inset shows the selected area electron diffraction (SAED) pattern for the as-synthesized PtSe_2_ nanosheets.

**Figure 4:**
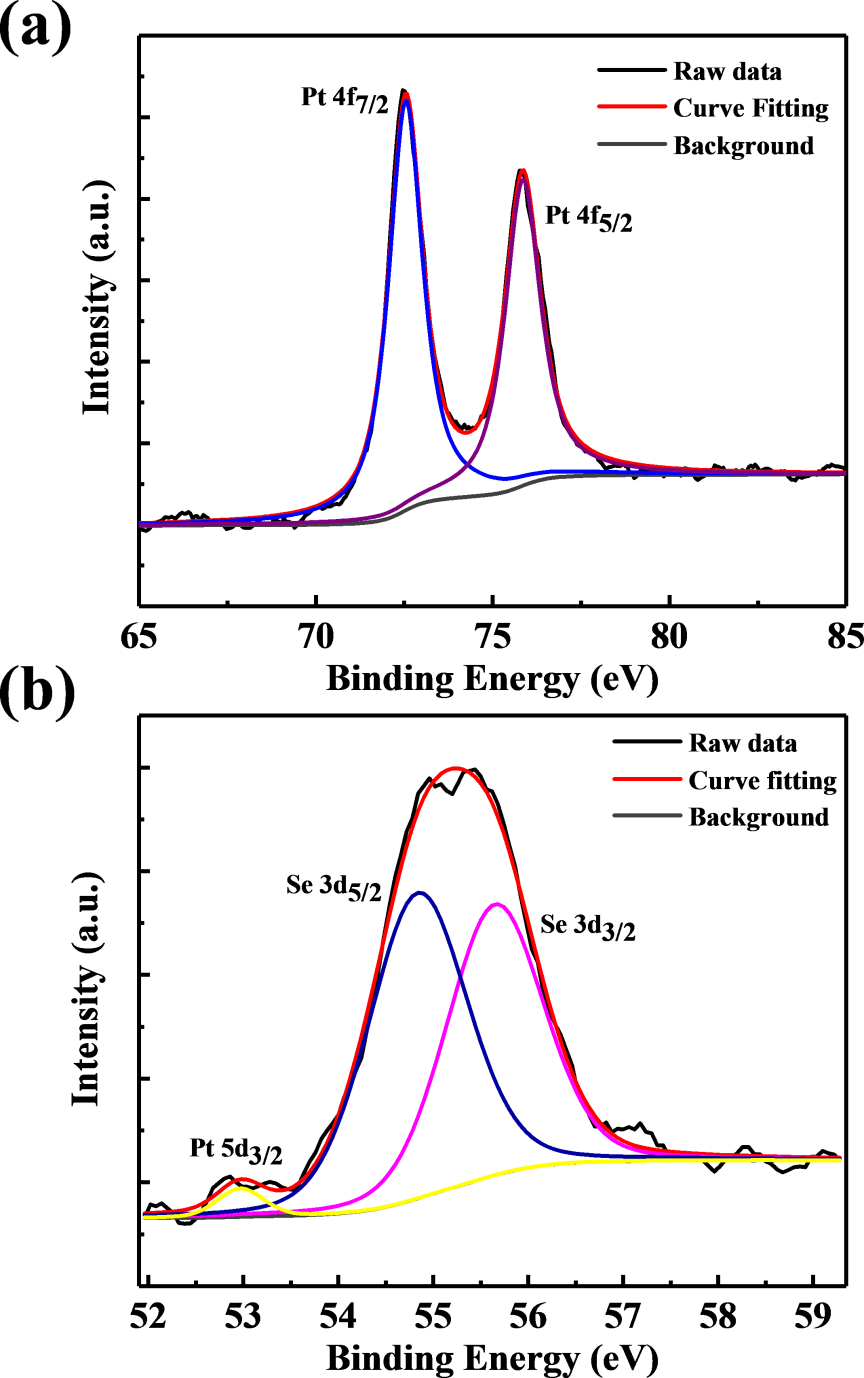
(a) Deconvoluted XPS spectra for Pt and (b) Se elements.

**Figure 5:**
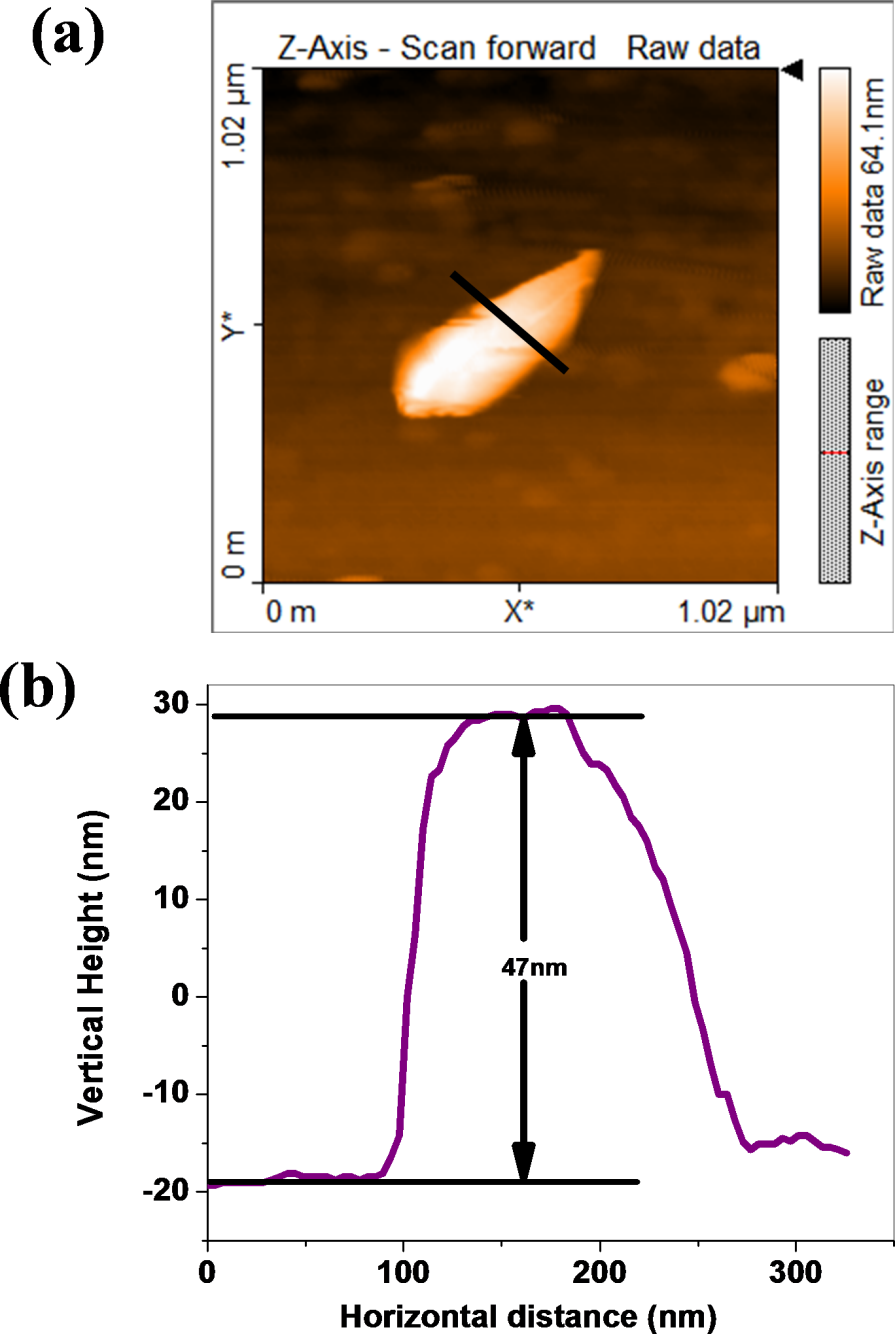
(a) AFM image and (b) AFM height profile plot for a PtSe_2_ nanosheet.

Morphological investigations were carried out using scanning electron microscopy (SEM). Figure 2a–c shows SEM images of few-layer PtSe_2_ with typical overlapping of multiple sheets on each other. Figure 2d shows an SEM image indicating a more transparent thin layer of PtSe_2_ stacked on each other, exhibiting the few-layer nature of the as-synthesized PtSe_2_ sample. Figure 3a–c shows the low-resolution TEM images of the as-synthesized PtSe_2_ sample clearly showing the sheet-like morphology with lateral dimension of ≈700 nm. Figure 3d shows a high-resolution TEM image of the PtSe_2_ nanosheets. The inset of Figure 3d shows the selected area electron diffraction pattern (SAED) which depicts the crystalline nature of the as-synthesized PtSe_2_ sample. The X-ray photoelectron spectroscopy (XPS) spectra of the Pt 4f and Se 3d regions acquired on a PtSe_2_ nanosheet sample were carried out on a film deposited on the Si substrate. The Figure 4a represents the fitted spectrum for Pt 4f_7/2_ and Pt 4f_5/2_ with binding energy 72.55 eV and 75.83 eV, respectively. Similarly, for Se, the binding energy spectrum can be fitted by Gaussian–Lorentzian curves shown in Figure 4b. The two peaks with binding energy 54.8 eV and 55.6 eV are observed for the 3d_5/2_ and 3d_3/2_ states, respectively. There is one more peak observed in the Se region with low intensity at 52.9 eV which corresponds to Pt 5d_3/2_ [24]. The thickness of the as-prepared PtSe_2_ nanosheets was calculated using atomic force microscopy (AFM). Figure 5a shows the AFM image which clearly shows that the lateral dimensions of the nanosheets are ≈700 nm. Figure 5b represents the corresponding height profile plot for the PtSe_2_ nanosheet with thickness found to be ≈47 nm.

### Temperature-dependent Raman spectroscopy of few-layer PtSe_2_ nanosheets

**Figure 6:**
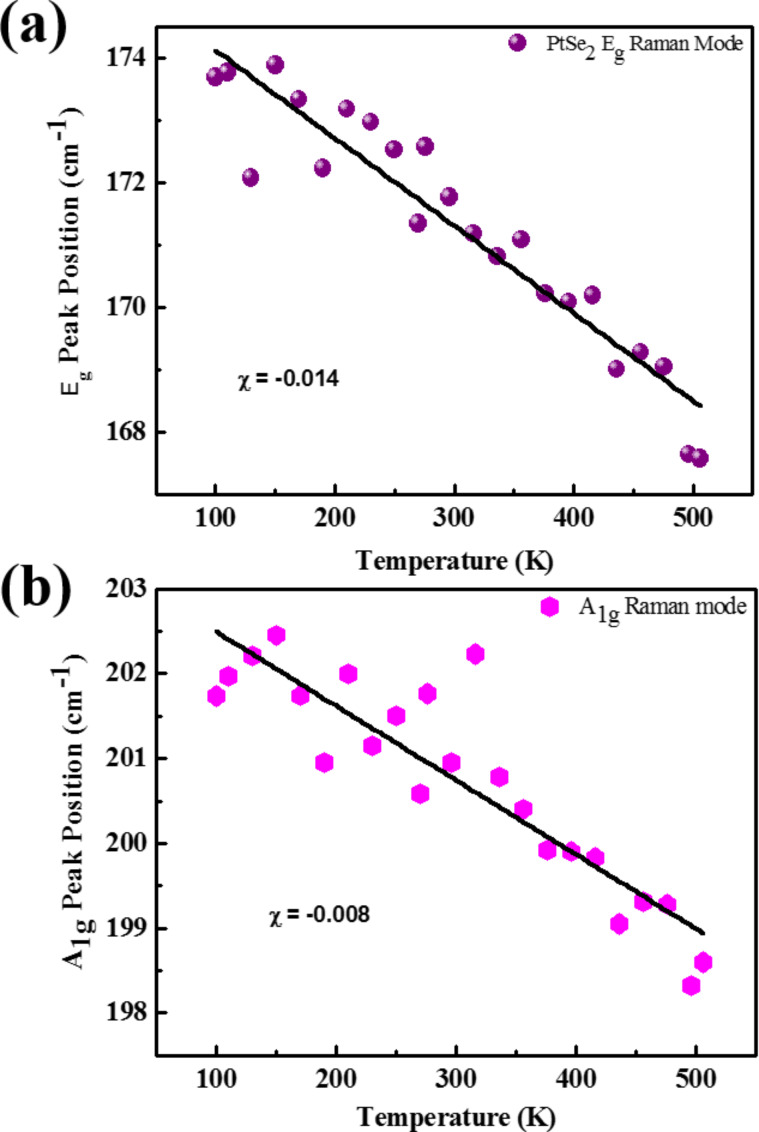
Temperature-dependent Raman spectra analysis for PtSe_2_ nanosheets for the (a) E_g_ mode and the (b) A_1g_ mode as a function of temperature.

The temperature-dependent Raman spectroscopy investigations of few-layer PtSe_2_ nanosheets were carried out between 100–506 K. The Raman spectra of the PtSe_2_ nanosheets at different temperatures are provided in Supporting Information File 1, Figures S2 and S3. The Raman mode E_g_ and A_1g_ as a function of temperature is shown in Figure 6a,b. It can be clearly seen that the position of the A_1g_ and E_g_ modes shifts to lower wavenumbers as the temperature increases from 100 K to 506 K. The Raman modes E_g_ and A_1g_ for PtSe_2_ behave linearly within the temperature range 100–506 K. Furthermore, it was observed that the full width half maximum (FWHM) increases with an increase in temperature. The peak positions in the Raman spectra were calculated by fitting the Lorentzian function to the A_1g_ and E_g_ modes. The temperature coefficient can be calculated by Equation 1 [29]:

[1] $$\omega \left(T\right)=\omega_{0}+\chi T\;,$$

where ω_0_ is the peak position of the A_1g_ and E_g_ mode at zero Kelvin, χ is the temperature coefficient of the A_1g_ and E_g_ modes, and ω is a Raman phonon frequency. The slope of the Raman modes vs temperature plot directly gives the value of the temperature coefficient and is given in Table 1. Further, it was clearly seen that the Raman peak position and peak broadening was affected by temperature. This change in Raman modes is mainly due to the contribution from the thermal anharmonicity. The Raman phonon frequency as a function of volume and temperature is given by Equation 2 [30]:

[2] $$\begin{array}{l} {\left(\frac{\partial \text{ln}\omega }{\partial T}\right)}_{P}={\left(\frac{\partial \text{ln}V}{\partial T}\right)}_{P}{\left(\frac{\partial \text{ln}\omega }{\partial \text{ln}V}\right)}_{T}+{\left(\frac{\partial \text{ln}\omega }{\partial T}\right)}_{V}\\ \;\;\;\;\;\;\;\;\;\;\;\;\;\;\;\;\;\;\;\;\;\;\;\;\;\;\;\;\;\;\;\;\;\;\;\;\;\;\;\;\;\;\;\;\;\;\;\;\;\;\;\;\;\;\;\;\;\;\;\;\;\;\;\;,\\ {\left(\frac{\partial \text{ln}\omega }{\partial T}\right)}_{P}=-\frac{\gamma }{K}{\left(\frac{\partial \text{ln}\omega }{\partial P}\right)}_{T}+{\left(\frac{\partial \text{ln}\omega }{\partial T}\right)}_{V} \end{array}$$

where γ is the volume thermal coefficient and *K* represents the isothermal volume compressibility. The first term on the right hand side, −γ/*K* (∂lnω/∂*P*)*_T_*, represents the volume contribution at a constant temperature. The second term, (∂lnω/∂T)*_V_*, represents the temperature contribution at constant volume. In single-layer TMDCs due to the direct band gap, the double resonance phenomenon is useful to explain the change in FWHM, intensity and the peak shift as a function of temperature. The double resonance phenomenon can be attributed to several process including absorption of an incident photon, creation of a hole pair, double scattering of a created hole pair by phonon, and recombination of an electron–hole pair with emission of phonon. The temperature coefficient for the E_g_ and A_1g_ modes was found to be −0.014 and −0.008, respectively. The nature of the temperature dependence of the Raman spectra of PtSe_2_ nanosheets is found to be similar in nature to that of graphene and other 2D materials such as MoS_2_, WS_2_, MoSe_2_, WSe_2_, BP, TiS_3_, multilayer graphene, and MoTe_2_ [29,31–34]. A comparison of the temperature coefficient values corresponding to various 2D materials are shown in Table 2. The value of Δω for both E_g_ and A_1g_ modes was found to be 6.11 cm^−1^ and 3.14 cm^−1^, respectively.

**Table 1:** Temperature coefficient values for the A_1g_ and E_g_ modes in a PtSe_2_ nanosheet sample.

Material	Raman modes	Temperature coefficient (χ)	∆ω (cm^−1^)

PtSe_2_ nanosheet	E_g_	−0.014	6.11
	A_1g_	−0.008	3.14

**Table 2:** Temperature coefficient values for various 2D materials.

TMDCs	Raman modes	Temperature coefficient (χ)	∆ω (cm^−1^)	Ref.

MoSe_2_	A_1g_	−0.0096	4.75	[29]
WSe_2_	A_1g_	−0.0071	3.81	[29]
MoS_2_	E_g_	−0.0136	8	[29]
	A_1g_	−0.0113	6.11	
WS_2_	E_g_	−0.0098	4.51	[29]
	A_1g_	−0.014	6.43	
black phosphorous	A_1g_	−0.008	4.39	[31]
	B_2g_	−0.013	8.14	
	A_2g_	−0.014	8.63	
TiS_3_	A_1g_	−0.022, −0.025, −0.024, −0.017	–	[32]
single-layer graphene	G	−0.0162	–	[33]
bilayer graphene	G	−0.0154	–	
MoTe_2_ (bilayer)	E’_2g_	−0.0116	–	[34]
	B’_2g_	−0.0181	–	
PtSe_2_	E_g_	−0.014	6.11	this work
	A_1g_	−0.008	3.14	

### Humidity sensor and photodetector based on few-layer PtSe_2_ nanosheets

**Figure 7:**
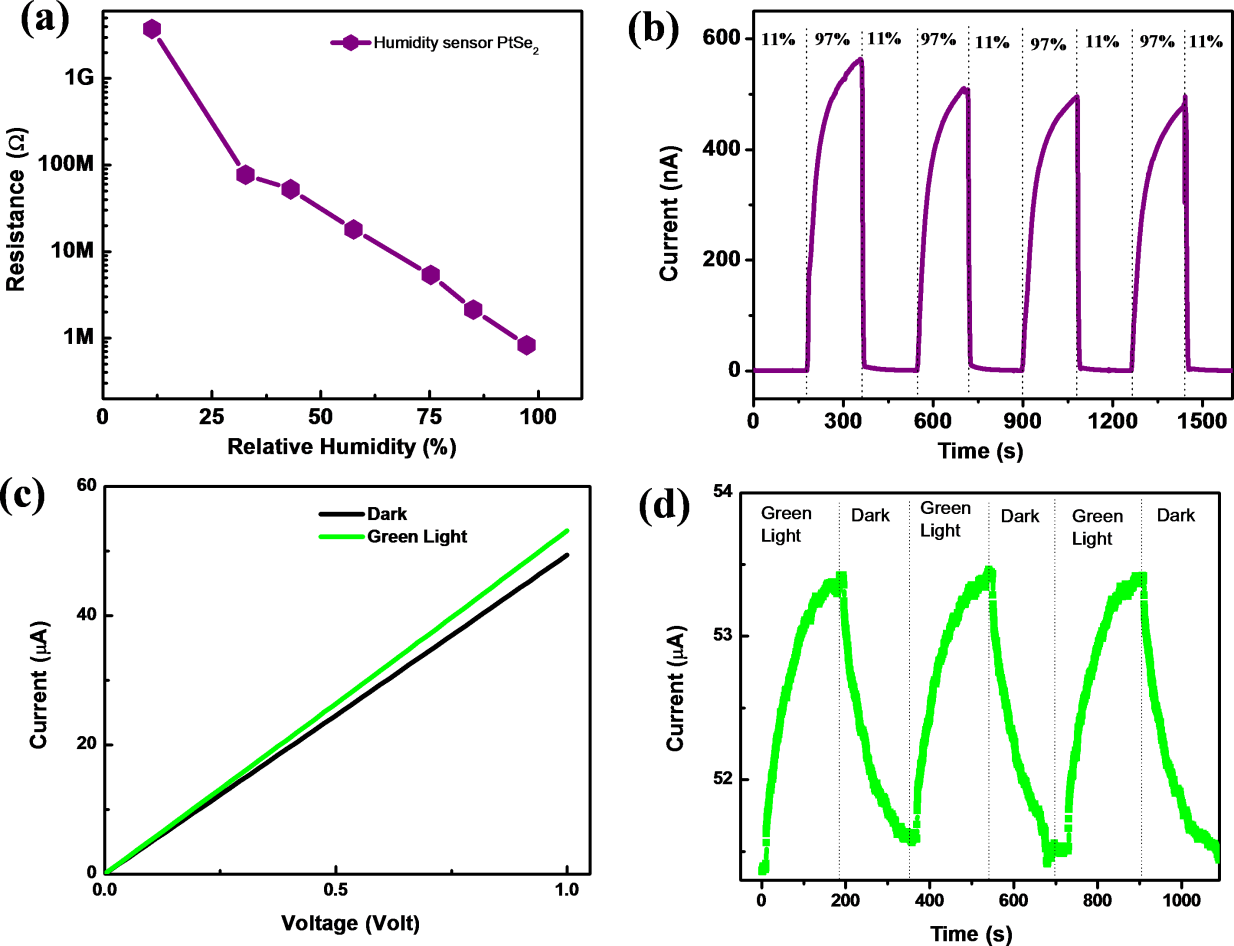
PtSe_2_ nanosheet based humidity sensor: (a) Typical resistance versus relative humidity plot and (b) current–time (*I*–*t*) plot taken after switching 11% RH and 97% RH. The photodetector application of PtSe_2_ nanosheets: (c) *I*–*V* in dark conditions and with green LED light and (d) typical *I*–*t* cycle when the LED is on and off, showing a favourable response.

Figure 7a shows the typical resistance of the sensor device vs relative humidity plot. The resistance is significantly decreased from 3.75 GΩ to 0.83 MΩ. The humidity sensing mechanism for the PtSe_2_ sensor can be explained as follows. When the PtSe_2_ nanosheet sensor device was exposed to water molecules/vapors, a charge transfer between the water molecules and the PtSe_2_ nanosheets occurs. This results in the decrease in resistance of the PtSe_2_ nanosheet sensor device with an increase in the relative humidity. The interactions among the water molecules (electron donor) and the PtSe_2_ nanosheets results in an enhancement in the conductivity of the sensor device, similar to that observed for other 2D materials such as SnSe_2_ [35], MoS_2_ [36], BP [26], and MoSe_2_ [37]. Figure 7b shows a typical current–time (*I*–*t*) plot where cycles of 11.3% and 97.3% RH levels were used to calculate the response and recovery time. The response and recovery time for the PtSe_2_-based humidity sensor device was found to be 118 s and 5 s, respectively. The advantage of the PtSe_2_-based humidity sensor device is its rapid recovery and its functionality at room temperature. Figure 7c shows a typical *I*–*V* plot in dark conditions and under green light illumination. Figure 7d shows the *I*–*t* plot for the photodetector based on PtSe_2_ nanosheets with a response time of ≈110 s and a recovery time of ≈129 s.

## Conclusion

In conclusion, we report on a wet chemistry method to grow PtSe_2_ nanosheets. The SEM and TEM analysis confirm the formation of PtSe_2_ nanosheets. Further, the XRD, Raman and SAED pattern results were used to analyze the crystal structure and to confirm the formation of the PtSe_2_ phase. Temperature-dependent Raman spectroscopy investigations were carried out on PtSe_2_ nanosheet films grown on Si substrates between 100–506 K. The temperature coefficient for the E_g_ and A_1g_ modes was found to be −0.014 and −0.008, respectively. A room temperature humidity sensor based on the PtSe_2_ nanosheets demonstrated an excellent recovery time of ≈5 s, indicating the great potential of PtSe_2_-based sensors for future nanoelectronics and sensor devices.

## Supporting Information

File 1 Additional figures.
