# Supplementary material for: Temperature-dependent Raman spectroscopy and sensor applications of PtSe2 nanosheets synthesized by wet chemistry
Source: Beilstein J Nanotechnol. 2019 Feb 13;10:467–74. doi: 10.3762/bjnano.10.46 (PMC6404413; doi:10.3762/bjnano.10.46)
Supplement: File 1 — Additional figures. [file Beilstein_J_Nanotechnol-10-467-s001.pdf]

BEILSTEIN JOURNAL OF NANOTECHNOLOGY

Supporting Information

for

Temperature-dependent Raman spectroscopy and sensor applications of $PtSe_2$ nanosheets synthesized by wet chemistry

Mahendra S. Pawar and Dattatray J. Late

Beilstein J. Nanotechnol. **2019,** *10,* 467–474. doi:10.3762/bjnano.10.46

Additional figures

License and Terms: This is a supporting information file under the terms of the Creative Commons Attribution License (http://creativecommons.org/licenses/by/4.0). Please note that the reuse, redistribution and reproduction in particular requires that the authors and source are credited.
The license is subject to the *Beilstein Journal of Nanotechnology* terms and conditions: (https://www.beilstein-journals.org/bjnano)

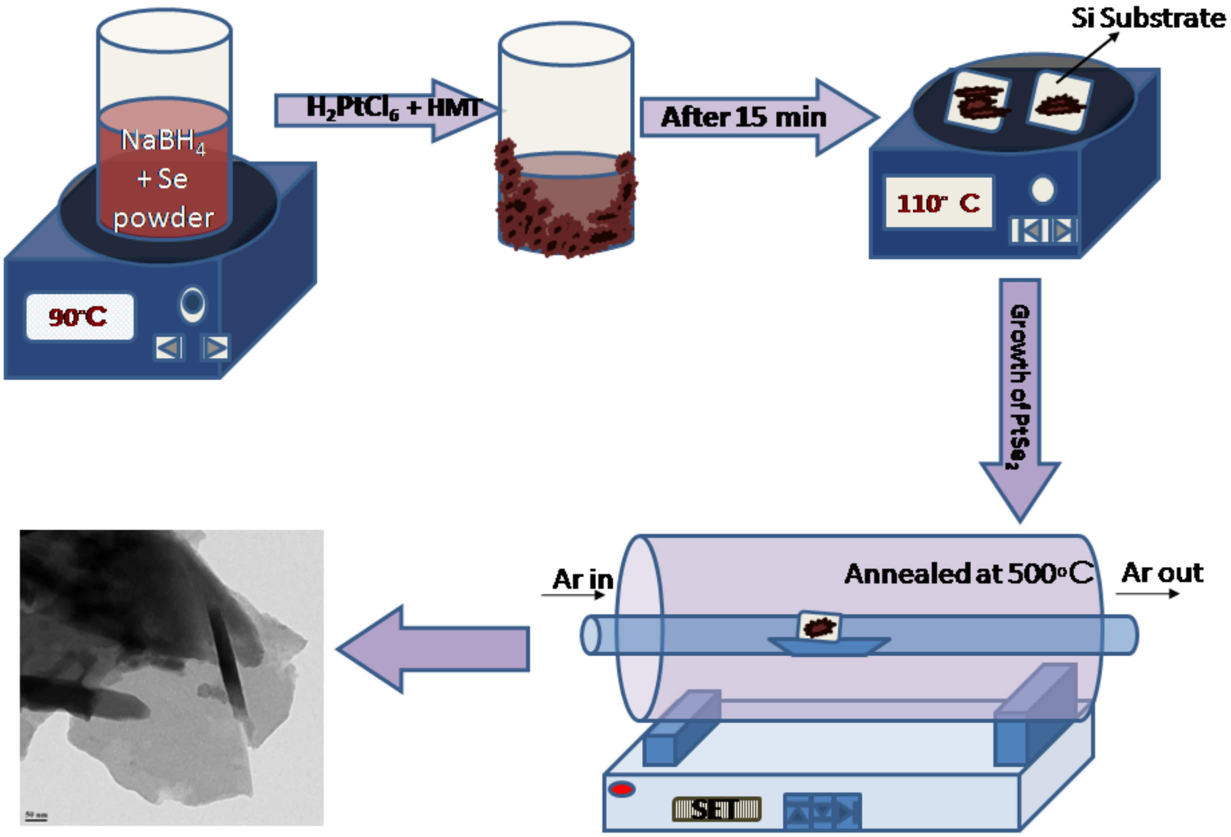

Figure S1: The schematic of $PtSe_2$ nanosheet synthesis steps.

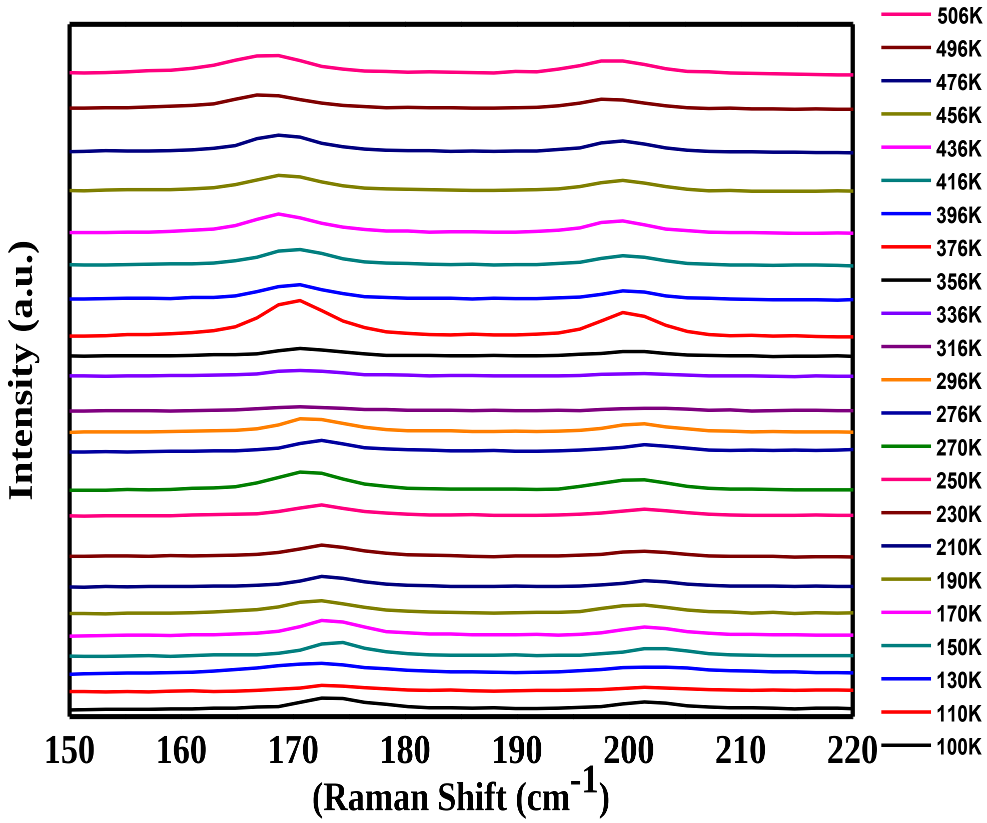

Figure S2: The Raman spectra of $PtSe_2$ nanosheets at different temperatures.

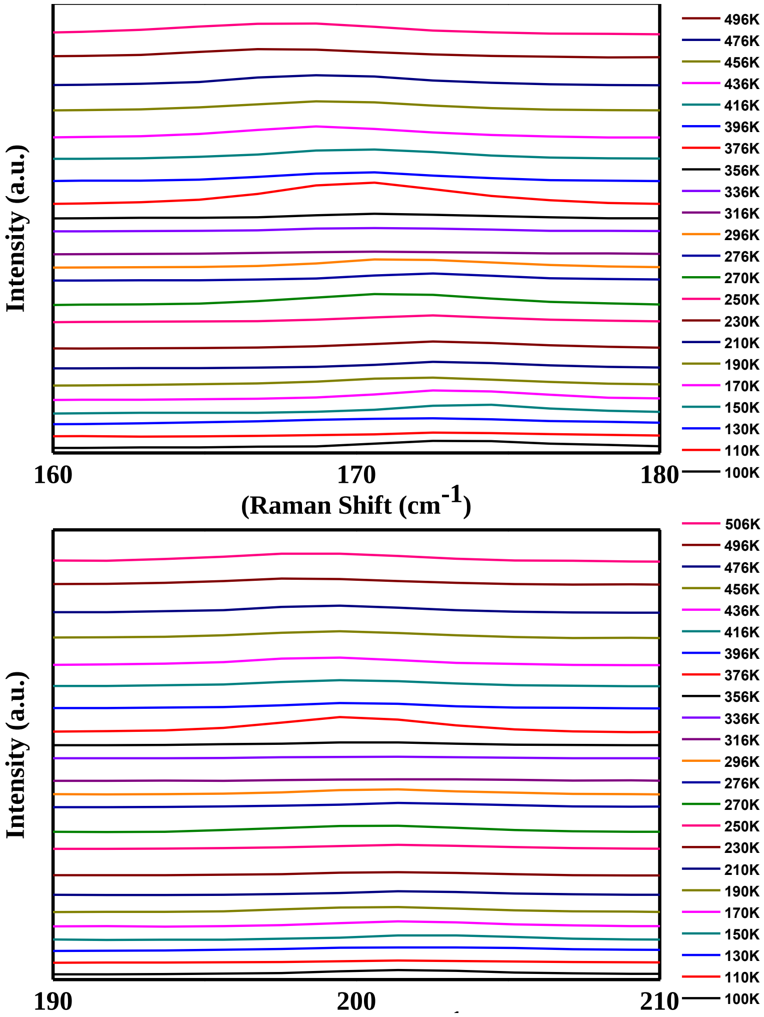

Figure S3: The normalized Raman spectra of $PtSe_2$ nanosheets at different temperatures for two modes.
